# Mini Review: Potential Applications of Non-host Resistance for Crop Improvement

**DOI:** 10.3389/fpls.2016.00997

**Published:** 2016-07-11

**Authors:** Seonghee Lee, Vance M. Whitaker, Samuel F. Hutton

**Affiliations:** Department of Horticultural Science, Gulf Coast Research and Education Center, Institute of Food and Agricultural Sciences, University of Florida, Gainesville, FLUSA

**Keywords:** broad-spectrum resistance, host resistance, *R*-gene, disease resistance, plant breeding

## Abstract

Plant breeding for disease resistance is crucial to sustain global crop production. For decades, plant breeders and researchers have extensively used host plant resistance genes (*R*-genes) to develop disease resistant cultivars. However, the general instability of *R*-genes in crop cultivars when challenged with diverse pathogen populations emphasizes the need for more stable means of resistance. Alternatively, non-host resistance is recognized as the most durable, broad-spectrum form of resistance against the majority of potential pathogens in plants and has gained great attention as an alternative target for managing resistance. While transgenic approaches have been utilized to transfer non-host resistance to host species, conventional breeding applications have been more elusive. Nevertheless, avenues for discovery and deployment of genetic loci for non-host resistance via hybridization are increasingly abundant, particularly when transferring genes among closely related species. In this mini review, we discuss current and developing applications of non-host resistance for crop improvement with a focus on the overlap between host and non-host mechanisms and the potential impacts of new technology.

## Introduction

Plant diseases pose a significant threat to global food security. Total world crop loss due to plant diseases and pests are estimated to be 20–40% each year (Oerke, 2006; Savary et al., 2012). Thus, plant breeding for disease resistance remains vital. The deployment of host resistance (*R*-genes) in crop cultivars typically involves introducing single or, preferably, multiple *R*-genes into susceptible genetic backgrounds via backcrossing or transformation. Unfortunately, *R*-gene mediated resistance is often not durable, since most plant pathogen populations have the adaptive potential to overcome resistance with time. A well-known example is stem rust (caused by *Puccinia graminis* Pers. f. sp. *tritici*) in wheat, the most important disease of wheat in many production regions, which can cause severe yield losses up to 70%. Pyramiding multiple stem rust *R*-genes has, for a long time, been a successful resistance management strategy (Singh et al., 2011). However, a new and aggressive race, Ug99, has been threatening many wheat growing regions (Pretorius et al., 2000). Due to the great diversity of pathogen races, identification of new sources of resistance and their transfer to advanced breeding germplasm are complex and challenging.

Non-host resistance (NHR) can be defined as the immunity of an entire plant species to all genetic variants of a non-adapted pathogen species. Since this type of resistance is typically both broad-spectrum and durable, NHR has considerable value for crop improvement and its practical application has been of recent interest. The majority of NHR studies have been performed in model plant species such as *Arabidopsis*, rice and *Nicotiana benthamiana*, and several important genes involved in NHR have been described (Lee et al., 2013; Senthil-Kumar and Mysore, 2013; Gill et al., 2015). However, despite the important role of NHR in plant defense, the mechanisms underlying NHR are still not clear in many cases. The potential application of NHR has been shown in multiple crops using genetically engineering (GM) methods. In crop species, examples of genetically engineered NHR applications include introduction of genes providing protection against fungal and bacterial pathogens, such as lettuce downy mildew, barley rust, strawberry gray mold and anthracnose, peach mildew, and tomato bacterial spot (Tai et al., 1999; Jafary et al., 2008; Jeuken et al., 2008; Qin et al., 2008; Zhang et al., 2009a; Lacombe et al., 2010; Sendín et al., 2012; Jiwan et al., 2013; Silva et al., 2015). Meanwhile, traditional breeding applications of NHR remain elusive but increasingly promising. Here, we address current approaches for application of NHR for disease resistance in crops and discuss potential avenues for future crop improvement.

## Basic Mechanisms of Non-Host Resistance and Their Overlap with Host Resistance

Plants defend themselves from pathogen attack through two major defense systems: the recognition of pathogen associated molecular patterns (PAMPs) by pattern recognition receptors (PRR) and effector-triggered immunity (ETI). The PRR type of resistance is characteristic of NHR and usually triggers multi-layered basal resistance mechanisms, which commonly include peroxisome-based biosynthesis, restriction of pathogen growth by nutrient limitation, papilla formation, and callose and lignin deposition (Ellis, 2006; Senthil-Kumar and Mysore, 2013). On the other hand, ETI is the main mechanism for host resistance (also known as *R*-gene mediated resistance) in which host R-proteins directly or indirectly recognize complementary pathogen effector proteins. As a result of the specific recognition in the host plant, a hypersensitive response (HR) involving cell death (or programmed cell death) often occurs, limiting or halting disease development.

To utilize NHR mechanisms for crop improvement, identification of the underlying genes is the first step. A number of studies have identified NHR genes effective against bacterial and fungal pathogens (Senthil-Kumar and Mysore, 2013; Cook et al., 2015; Gill et al., 2015). Thus far, many of the identified genes involved in NHR have multi-functional roles including plant development and stomatal regulation, and are not only important for the non-host defense signaling pathway itself, but also in basic plant metabolism (Rojas et al., 2012; Lee et al., 2013; Senthil-Kumar and Mysore, 2013; Dong et al., 2015). For example, glycolate oxidase and proline dehydrogenase modulate ROS mediated signal transduction pathways in response to various environmental stresses, and these enzymes are involved in NHR against bacterial pathogens (Rojas et al., 2012; Senthil-Kumar and Mysore, 2013). Heterotrimeric G-proteins consist of three distinct subunits, Gα, Gβ, and Gγ that are well conserved in all eukayotes and important for the cell signaling and plant development. It has been reported that the mutation of G-proteins compromises NHR against fungal and bacterial pathogens (Lee et al., 2013; Liang et al., 2016).

Non-host resistance can be divided into two categories based on the presence and absence of visual cell death: Type I and Type II NHR. Type I NHR does not show any visible cell death symptoms while type II NHR shows localized hypersensitive response (HR) cell death in response to non-host pathogens. This cell death is mediated through the generation of reactive oxygen species (ROS) and shares similarities with effector-triggered immunity, the main mechanism for host resistance. The plant defense-related genes responsible for initial defense response (pathogenesis-related protein genes, oxidative burst-associated genes, and cell defense genes) are similarly expressed in both Type II NHR and host resistance in tobacco (Oh et al., 2006), suggesting overlapping defense signaling pathways between non-host and host resistance.

Non-host resistance against pathogens of distantly related hosts usually involves complex, multi-layered, quantitatively inherited mechanisms, including those mentioned above. However, NHR against pathogens of more closely related species can involve mechanisms more similar to host resistance. For instance, there is increasing evidence that stacked *R-*genes can confer non-host status (Mysore and Ryu, 2004), specifically NB-LRR type genes (Schulze-Lefert and Panstruga, 2011). For example, the recognition of pathogen avirulence protein (Avr) by non-host plant species has been reported in maize, soybean and pepper. The maize resistance gene *Rxo1* can recognize the rice bacterial streak pathogen (Zhao B. et al., 2004, 2005). The pepper bacterial spot resistance gene *Bs2* recognizes the *Xanthomonas* pathogen of tomato (Tai et al., 1999). Indeed, the line between host and non-host can be significantly blurred via a range of responses between purely compatible and incompatible interactions, as in the case of cereal rusts (Bettgenhaeuser et al., 2014).

As we will see in the next section, transgenic approaches have been most effective for transfer of basal defense mechanisms to distantly related host species, but traditional inheritance studies and breeding strategies have been most feasible for transfer of non-host mechanisms between more closely related species.

## Applications of Non-Host Resistance for Crop Diseae Resistance: Genetic Engineering Approaches

It is evident that the components required for NHR can be genetically engineered to control host-adapted pathogens in crops. Strawberry is an excellent example of an economically important fruit crop worldwide that has also been recognized as a suitable model plant species for the Rosaceae family. The *Arabidopsis NPR1* (*Non-expresser of PR genes 1*) gene regulates the onset of systemic acquired resistance through the salicylic acid defense pathway and via defense genes such as *PR* (pathogenesis-related) genes for plant immunity. The ectopic expression of *Arabidopsis NPR1* in diploid strawberry (*Fragaria vesca*) increases resistance to anthracnose (*Colletotrichum* spp.), powdery mildew (*Podosphaera aphanis*), and bacterial angular leaf spot (*Xanthomonas fragariae*) (Silva et al., 2015). In another example, allelic variation for powdery mildew resistance genes, *MILDEW-RESISTANCE LOCUS* (*MLO*), is highly diverse among monocotyledonous and dicotyledonous plant species. Barley, in particular, has highly effective immunity against powdery mildew fungi, and this immunity is achieved by loss-of-function mutant alleles of the barley MLO gene (Humphry et al., 2006). It is suggested that the resistance present among different plants is due to similar *mlo*-based immunity, natural loss-of-function polymorphisms in *MLO* gene (Piffanelli et al., 2004; Bai et al., 2005). For example, the *PpMlo1* (*Prunus perisica* Mlo) of peach has been identified for powdery mildew resistance and shares a similar functional mechanism for resistance in the Rosaceae (Jiwan et al., 2013). The antisense expression of the peach *MLO* gene (*PpMlo1*) in octoploid strawberry conferred NHR to strawberry powdery mildew (*Podosphaera aphanis*) (Jiwan et al., 2013).

The transfer of *R*-genes from a distantly related host into another host has been successfully used to control major plant diseases. Presumably, this approach is often successful due to the divergence in resistance motifs from different plant species. In that sense, these examples approximate the NHR among more closely related plant species that may be controlled at least partly by ETI mechanisms. For example, bacterial spot of tomato and pepper (caused by several *Xanthomonas* spp.) is a devastating disease in warm and humid environments, such as Florida. But whereas pepper breeders have enjoyed some success against the pathogen through the development of resistant cultivars (Horvath et al., 2015), no resistant cultivars have been deployed in tomato against bacterial spot disease, despite decades of conventional breeding efforts (Sim et al., 2015). The transfer of the *Bs2 R*-gene from pepper to tomato results in a very strong resistance to most species and races of bacterial spot in tomato (Scott et al., 2011; Horvath et al., 2012). But pathogen strains have already emerged which have mutations in their *avrBs2*, prompting tomato researchers to pursue more durable lines of defense. One current approach for bacterial spot resistance in tomato is expression of the *Bs2* gene along with the *Arabidopsis EFR* gene, a leucine rich repeat receptor-kinase that recognizes a bacterial protein. A similar receptor does not exist in tomato. Transgenic tomato plants with *Arabidopsis EFR* acquired broad spectrum bacterial resistance in tomato, including to bacterial spot (Lacombe et al., 2010). Constitutive activation of ROS production or defense-gene expression was not found in transgenic tomato plants expressing *EFR*. Also, more importantly, no growth or development defects were observed in the transgenic tomato plants. Taken together, this result is a fascinating example of a combination of PRRs with R proteins conferring elevated, broad-spectrum resistance to a number of bacterial genera when expressed in tomato.

Cereal crops belong to the same grass family, but their diseases are specific to species. Yet the *Rxo1* locus of maize confers resistance to all races of the rice bacterial leaf streak pathogen *Xanthomonas oryzae pv. oryzicola* (Zhao B.Y. et al., 2004, 2005). There is no major gene available for bacterial streak resistance in rice, though this is an important disease in Asia. Transgenic rice plants expressing *Rxo1* showed strong resistance against *X. o*. pv. *oryzicola* (Zhao et al., 2005). Interestingly, this transgenic rice is resistant to another bacterial spot pathogen, *Burkholderia andropogonis*, which is a distantly related pathogen of rice. The mechanism for *Rxo1*-mediated resistance is not clear, but this is an excellent example of a single NHR locus conferring broad-spectrum resistance.

Citrus greening (or Huanglongbing, HLB) is a serious phloem-limited disease caused by a vector-transmitted bacterial pathogen, *Candidatus* spp. There is no resistant citrus cultivar available; thus, extensive research on genetically modified alternative resistances has been conducted. Once again, the *NPR1* gene from *Arabidopsis* thaliana has been transformed into two commercial sweet orange cultivars, Hamlin and Valencia, introducing enhanced disease resistance to HLB (Dutt et al., 2015). The endogenous expression of *AtNPR1* constitutively down-regulated the signaling pathway for plant immunity in the transgenic citrus and helped to maintain resistance to HLB for a long period under high disease pressure in field. Since the plant *NPR1* gene can promote the SA pathway, the continuous expression of *AtNPR1* in citrus may also inhibit HLB bacteria colonization and multiplication in the infected phloem. This finding would be tremendously important for the citrus industry, and would provide evidence of a practical application of NHR for controlling a major citrus disease.

The use of conventional breeding approaches to transfer the genetic components involved in NHR across species is, in many cases, difficult or maybe even impossible with current available methods. Although genetic engineering provides a solution this this obstacle, social barriers to the adoption of genetically modified organisms (GMOs) remain quite strong, particularly for the majority of freshly consumed commodities. In such cases, the recent gene-editing technology, i.e., clustered, regularly interspaced, short pallindromic repeats (CRISPR), could be effective for the transfer of single or even multiple target genes for NHR to distantly related plant species, while potentially avoiding US regulation for GMOs (Waltz, 2016).

## Applications of Non-Host Resistance for Crop Diseae Resistance: Traditional Breeding Approaches

The first challenge in breeding for NHR mechanisms, is the identification of NHR loci that can be transferred to the host species of interest. This, of course, necessitates that closely related non-host species are interfertile with the host species of interest. While it may sound simple, one of the first possibilities for non-host gene identification is to screen the natural diversity of related species accessions to identify rare individuals that have become infected. This would then be followed by crossing and inheritance studies within the non-host species to identify the causal loci (Shafiei et al., 2007). Atienza et al. (2004) relates a similar strategy in barley, a marginal host to several cereal rusts, in which natural susceptible mutants were crossed to non-host lines (Atienza et al., 2004). They found that immunity is conferred by a number of quantitative trait loci (QTL) with overlapping specificities to rust fungal species. In a similar strategy, the non-host may be subjected to mutation screening and/or gene silencing and observed for induced susceptibility, as in the original *PEN* gene studies in *Arabidopsis* (Collins et al., 2003).

In all these examples, the common thread is the identification of susceptible mutants in non-host species that allow the induction or identification of NHR loci. The genes of interest, if suitable for crop improvement, can then be transferred to the host species via hybridization with the non-host species if possible. A related, and more ideal, strategy would be to hybridize interfertile host and non-host species and perform QTL mapping studies, as has been accomplished for the *Bremia*–*Lactuca* interaction and rye-wheat translocation (Masoudi-Nejad et al., 2002; Jeuken et al., 2008; Zhang et al., 2009b; den Boer et al., 2014). This allows the identification of NHR loci and the transfer of the target loci to the host species in the same step. As an example, the lettuce downy mildew (causal pathogen *Bremia lactucae*) non-host *Lactuca saligna* was crossed to host species *L. sativa*, and backcross inbred lines were developed to stack alleles at four recessive NHR QTL. Three stacked QTL were sufficient to confer complete resistance to lettuce downy mildew. Interestingly, the three QTL showed epistatic interactions with increased resistance levels (Zhang et al., 2009a), suggesting some synergy among the mechanisms controlled by the three loci.

The utilization of NHR genes in breeding carries with it one of the same concerns as host resistance breeding. For host resistance, the use of single *R* genes can be risky due to the potential evolution of pathogenic races and lack of durability. For this reason gene pyramiding is often pursued with the intent to enhance the durability of resistance. For NHR, the same scenario could be possible. For example, the *Yr9* resistance gene transferred from rye to wheat via wide hybridization was reported to have eventually broken down under pathogen pressure (Yang et al., 2014; Tian et al., 2016). Thus, sources of NHR that are polygenic, such as in the *Bremia*–*Lactuca* interaction, are likely to be more durable if indeed they represent multiple layers of resistance or redundant mechanisms. At any rate, it is suggested that deployment of single genes for NHR should be avoided, as well as for host resistance (Bettgenhaeuser et al., 2014). For example, bacterial leaf blight (*X*. *o*. pv. *oryzae*) and bacterial leaf streak (*X*. *o*. pv. *oryzicola*) are the most important bacterial pathogens in rice, and the use of single, conventionally bred resistance genes has not been effective for maintaining breeding gains. But a recent study demonstrated that a cultivar pyramided with the *R* gene *Xa23* and the *Rxo1* gene from maize showed a high level of resistance to both bacterial diseases in rice. In this case, the pyramiding involved both a host gene transferred through conventional hybridization and a non-host gene transferred by transgenic means.

## Conclusion and Perspective

In the last decade, numerous studies have identified genetic components regulating NHR. Genetic systems that involve the recognition of the most essential chemical components of plant pathogens should provide the greatest durability of resistance. Although the broad-spectrum resistance and potential durability of NHR may provide value over traditional *R*-gene breeding, regular application of NHR-based disease resistance is still to come. Genetic engineering is advantageous for transferring PRR resistance components among distantly related species, particularly those resistances that are robustly multi-layered. Due to the complexities of deregulation and persistent social barriers to genetically engineered crops, genome-editing technologies such as CRISPR may provide an alternative mode for the transfer of NHR, particularly via targeted modification of pathways that are the most conserved among plant species. Meanwhile, for more closely related and interfertile species, mutation screening and traditional hybridization can be employed for conventional breeding, keeping in mind that deployment of single non-host genes may not be sufficient for durability. As our basic understanding of NHR mechanisms advances in both model and crop systems, our agricultural disease resistance toolbox should only become larger.

## Author Contributions

SL organized and prepared all this manuscript SH and VW contributed for writing and reviewing the major part of the manuscript.

## Conflict of Interest Statement

The authors declare that the research was conducted in the absence of any commercial or financial relationships that could be construed as a potential conflict of interest.
